# Pharmacological Effects of *Rosa Damascena*

**Published:** 2011

**Authors:** Mohammad Hossein Boskabady, Mohammad Naser Shafei, Zahra Saberi, Somayeh Amini

**Affiliations:** 1 *Department of Physiology, School of Medicine and Pharmaceutical Research Centre, Mashhad University of Medical Sciences, Mashhad, Iran*; 2 *Department of Horticulture, College of Agriculture, Ferdowsi University of Mashhad, Mashhad, Iran*

**Keywords:** Damask Rose, Essential oil, Pharmacological properties, damascena, Rose water

## Abstract

*Rosa damascena* mill L., known as Gole Mohammadi in is one of the most important species of Rosaceae family flowers. *R. damascena* is an ornamental plant and beside perfuming effect, several pharmacological properties including anti-HIV, antibacterial, antioxidant, antitussive, hypnotic, antidiabetic, and relaxant effect on tracheal chains have been reported for this plant. This article is a comprehensive review on pharmacological effects of *R. damascena*.

Online literature searches were performed using Medline, medex, Scopus, and Google Scholar websites backed to 1972 to identify researches about *R. damascena*. Searches also were done by going through the author's files and the bibliographies of all located papers.

## Introduction

*Rosa damascena* mill L, commonly known as Damask rose (1), is known as Gole Mohammadi in Iran (2). It is one of the most important species of Rosaceae family. Rosaceae are well- known ornamental plants and have been referred to as the king of flowers (3,4). At present time, over 200 rose species and more than 18000 cultivars form of the plant have been identified (5). Apart from the use of* R. damascena* as ornamental plants in parks, gardens, and houses, they are principally cultivated for using in perfume, medicine and food industry (6). However,* R. damascena* is mainly known for its perfuming effects (7). The rose water were scattered at weddings to ensure a happy marriage and are symbol of love and purity and are also used to aid meditation and prayer. 

There is a strong bond between Iranians and this plant. Its popularity is not only because of the medicinal effects but also is due to holy beliefs about it. People call this plant Flower of Prophet Mohammed (Gole mohammadi), because they believe its nice aroma reminds them of prophet Mohammad (8).

At the present time, this plant is cultivated in Iran (especially in Kashan) for preparing rose water and essential oil (9, 10). Because of the low oil content in *R. damascena* and the lack of natural and synthetic substitutes, essential rose oil of this plant is one of the most expensive ones in the world markets (11). 

The *R. damascena* has also been used for medicinal purposes (12). Various products and isolated constituents from flowers, petals and hips (seed-pot) of this plant have been studied in a variety of *in vivo* and *in vitro *studies. However, there are not any reviews to collect pharmacological effects of *R. damascena* in the present time. Therefore, in this review we collect and discuss important pharmacological effects of *R. damascena* that recently have been published in numerous studies.

***Morphology***

**Figure1.**
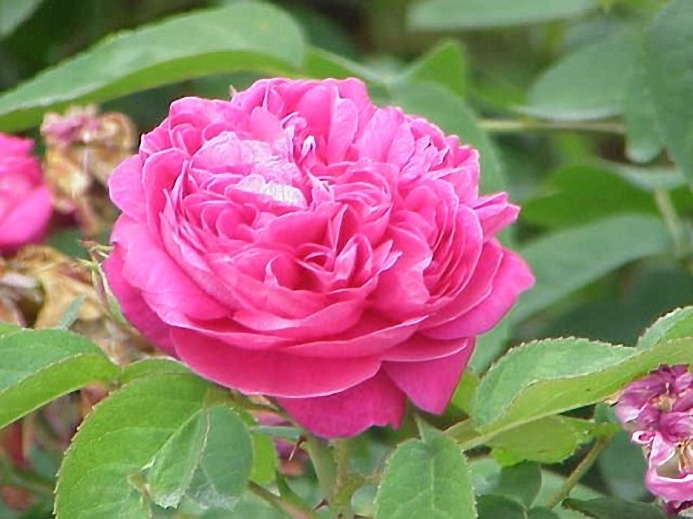
The plant of R*. damascena*

*R. damascena* is a perennial bushy shrub reaching approximately 1 to 2 meters in height with large, showy and colorful flowers. The leaves are imparipinnate and compound with 5-7 leaflets (13,14) (Figure 1).

Its life span is up to 50 and economic period is about 25 years. Gestation period is three years for attaining economic production level. Its propagation is mostly by cutting and using Suckers but micropropagation is a developing propagation method for this plant in Iran (8). 

***History***

There are evidences that Rosaceae family is an ancient plant (8, 9,15). Some fossils of rose are found in America that are 30 million years old (15). The origin of Damask rose is the Middle East and some evidences indicate that the origin of rose water is Iran, but the origin of its fragrant oil and extracts is Greece (16). This plant is cultivated in all over the world including Iran, Europe, Bulgaria, Turkey and India (17). The major cultivation areas of* R. damascena* in Iran are Kashan, Fars and Azerbaijan, among them Kashan is the most famous one (8). 

There are many evidences that cultivation and consumption of *R. damascena* in Iran has a long history and Iran is one of its origins (18). It is believed that the crude distillation of roses for the oil was originated from in the late 7th century AD, and spread to the provinces of later in 14th century. Iran was the main producer of rose oil until the 16th century and exported it to all around the world (19-21).

***Traditional uses***

The most therapeutic effects of *R. damascena* in ancient medicine are including treatment of abdominal and chest pain, strengthening the heart (22), treatment of menstrual bleeding and digestive problems (23), and reduction of inflammation, especially of the neck (24). North American Indian tribes used a decoction of the root of *R. damascena* plant as a cough remedy to ease children’s cough (13). This plant is also used as a gentle laxative (16). Rose oil heals depression, grief, nervous stress and tension. It helps in the reduction of thirst, healing old caugh, special complaints of women, wound healing, and skin health. Vapor therapy of rose oil is helpful for some allergies, headaches, and migraine (16,25). 

***Products***

There are different products from* R. damascena* in the world. The major products are as below. 

*Rose water*

It is an abundant product of *R. damascena* in which contains 10-50% rose oil. The most usage of Rose water is in religious ceremonies. It is used in mosques especially at mourning ceremonies, to calm and relax people. The highest quality rose water is produced in Kashan. Kaaba (God House) in , is washed yearly by unique and special rose water of Kashan. Rose water is also of high value in the food industry and some special foods are prepared using this product (8).

*Rose oil*

It is a volatile oil obtained by distillation of the fresh flowers of *R. damascena*. The chief producing countries are , , and , but not a major product in . The oil is prepared in copper alembic stills by the peasant or in large factories under careful scientific control. Some 3000 parts of flowers yields only one part of oil. The oil is very expensive and very liable to adulteration. The oil is, pale, yellow, and semisolid. The portion which is solid at ordinary temperatures forms about 15-20% and consists of odourless stearoptene containing principally saturated aliphatic hydrocarbons (C14-C23 normal paraffins) (8, 26). Because of the low oil content in *R. damascena* and the lack of natural and synthetic substitutes, essential rose oil is one of the most expensive ones in the world markets (11).

*Dried flowers*

 Two kinds of dried flowers are produced in . A) Dried bud which is mostly for export. B) Dried petals for different purposes; its major use is for eating, as it can solve problems with digestive system. Some Iranians eat it with yogurt. Another reason for drying petals is to store them when distilleries cannot accept the whole produced flower anymore. They use them later for distillation (8,16).

*Hips*

Both dried and fresh hips of *R. damascena *processed or not processed, are used in Iran (8). 

*Other products*

Other different products are including hydrosol, absolute, ethanolic, aqueous, and chloroformic extractions from flowers, petals, and hips (seed-pot) of this plant. In comparison with rose oil, hydrosol and absolute are less expensive. The ethanolic, aqueous, and chloroform extracts are also prepared for research purposes (10).

***Chemical composition***

Several components were isolated from flowers, petals and hips (seed-pot) of *R. damascena* including terpenes, glycosides, flavonoids, and anthocyanins (27-30). This plant contains carboxylic acid (31), myrcene (32), vitamin C (13), kaempferol and quarcetin (33). Flowers also contain a bitter principle, tanning matter, fatty oil and organic acids (34). Loghmani-Khouzani *et al* (2007) found more than 95 macro- and micro-components in the essential oil of *R. damascena* from the Kashan regions of . Among them, eighteen compounds represented more than 95% of the total oil. The identified compounds were; β-citronellol (14.5-47.5%), nonadecane (10.5-40.5%), geraniol (5.5-18%), and nerol and kaempferol were the major components of the oil (2). Analyses of rose absolute showed that phenyl ethylalcohol (78.38%), citrenellol (9.91%), nonadecane (4.35%) and geraniol (3.71%) ethanol (0.00-13.43%), and heneicosane were the major compounds (35). In another study, the composition of rose was phenyl ethylalcohol (72.73–73.80%), citrenellol (10.62–11.26%), nerol (2.42–2.47%), and geranial (5.58–5.65%) (36). Hydrosol was also found to contain four constituents; geraniol was the major compound (30.74%) followed by citrenellol (29.44%), phenyl ethylalcohol (23.74%), and nerol (16.12%) (9, 35).

The medicinal functions of Rosaceae are partly attributed to their abundance of phenolics compound. Phenolics possess a wide range of pharmacological activities, such as antioxidants, free-radical scavengers, anticancer, anti-inflammatory, antimutagenic, and antidepressant (12, 38-42).

## Pharmacological studies

Different pharmacological effects of *R. damascena* are as follows (Table 1).

***Neuropharmacolgical effects***

Several Pharmacological studies have been performed on *R. damascena *to evaluate their effects on the central nervous system (CNS). The effects of this plant on CNS are extensive. 

Ethanolic extract of the flowering tops of *R. damascena *has been shown to possess a potent depressant activity on CNS in mice (34). Some of these effects that evaluated are hypnotic, anticonvulsant, anti-depressant, anti-anxiety, analgesic effects, and nerve growth that are discussed below. 

*Hypnotic effect*

One of the effects of *R. damascena* on central nervous system is its hypnotic effect. The ethanolic, aqueous and chloroformic extracts from *R. damascena* were used for hypnotic effect in mice. The ethanolic and aqueous extracts in doses of 500 and 1000 mg/kg significantly increased the pentobarbital induced sleeping time in mice which was comparable to diazepam. However, the chloroformic extract has not shown to have hypnotic effect (43,44). 

In another study, the hypnotic effects of three fractions (ethyl acetate, aqueous and n-butanol fractions) of this plant were evaluated. It has been shown pentobarbital induced sleeping time increased by these fractions. Among these fractions, the ethyl acetate fraction has the best hypnotic effect. The ethanol crude extract of *R. damascena *and its fractions were also investigated in mice. It was shown that they can prolong the pentobarbital induced sleeping time comparable to diazepam (45). Although the hypnotic effect of the extracts and fractions of *R. damascena *have been shown but the mechanism(s) of hypnotic effect of this plant was (were) not clarified. *R. damascena *contains several components such as flavonoids and terpenes (13, 46). There are evidences that these compounds have hypnotic effect (44,47). Therefore, it is suggested that these compounds may be responsible for the hypnotic effect of *R. damascena*. Flavonoids have been shown to have anxiolytic and/or antidepressant activity in numerous studies (18, 43,44). It can be suggested that flavonoids of the *R. damascena *contribute to the hypnotic effect. This effect has been ascribed to their affinity for the central benzodiazepine receptors (44). Noguerira and Vassilieff have shown that the other genuses of Rosaceae family exert their hypnotic effect through GABAergic system (48). Therefore, this system is probably another mechanism involved in the hypnotic effect of *R. damascena*.

*The* *analgesic* *effect*

The analgesic effect of *R. damascena *is also reported. In a study, the effect of aqueous, ethanolic and chlorphormic extracts in mice on hot plate and tail flick was evaluated and only ethanolic extract showed analgesic effect (49). The analgesic activity of hydroalcoholic extract and essential oil of *R. damascene* in acetic acid formalin and tail flick tests in mice demonstrated that essential oil of the plant failed to show any analgesic effect. However, hydroalcoholic extract has a potent analgesic effect in acetic acid and formalin tests and no effect on tail flick test (50). 

Based on analgesic effect of hydroalcoholic and ethanolic extracts, it is suggested that ingredients of the plant that are not soluble in water may be responsible for observed analgesic effect. Therefore, it is suggested quercetin and kaempferol which are not soluble in water may be responsible for this effect (49,51).

Recently, it has been reported that antioxidants reduce pain in formalin test (52). It has been reported that *R. damascena *contains flavonoid (2, 53,54). Therefore, it seems that these compounds have some role in the analgesic effect of the plant. In tail flick test, essential oil and hydroalcoholic extract could not exert any antinociceptive activity but ethanolic extract could affect tail flick test. The mechanisms of these effects are not completely known and further studies are needed to find out the exact mechanism.

*Protective effects on neuritic atrophy*

*R. damascena* has beneficial effects on the brain function such as treatment of dementia. Awale *et al* (2009) showed neurite outgrowth activity of rose extract (55). They found that the chloroformic extract of the *R. damascena* significantly induced the neurite outgrowth activity and inhibited the amyloid β (Aβ) (55). Aβ is thought to be a major pathological cause of Alzheimer. Aβ (25-35) is major fragment of full peptide of Aβ and can be produced in the brains of Alzheimer’s patients. Aβ (25-35) caused neural cell death, neuritic atrophy, synaptic loss, and memory impairment (56-61).

An active constituent of chloroform extract of *R. damascena* was isolated which is a very long polyunsaturated fatty acid (VLFA) having molecular formula C_37_H_64_O_2_. This isolated compound protected atrophy induced by Aβ (25-35) and displayed strong neurite outgrowth activity. The effect of this compound on length of dendrite in the treated cells was comparable to those of nerve growth factor (NGF) (55). Therefore *R. damascena* may have beneficial effect in patients suffering from dementia.

*Anticonvulsant effect*

The essential oil of *R. damascena* in acute pentylenetetrazole (PTZ)-induced seizure in rats, delays the start of epileptic seizures and decrease the duration of tonic-clonic seizures (stage 4) (62,63). In chronic model of PTZ-induced seizure, this plant also caused prolongation of latent periods before tonic-clonic generalized seizures (62). 

Injection of essential oil 30 min before amygdale electrical kindling also reduced appearance of 1st, 2nd, 3rd, 4th, and 5th stages of seizure and could reduce the time after discharge duration. It is suggested that essential oil of *R. damascena* retarded the development of behavioral seizures in amygdale electrical kindling and possesses the ability to counteract kindling acquisition (63). 

The mechanism(s) of these effects of *R. damascena* cannot be explained by the observed results. However, authors suggested that the flavonoieds maybe involved in this effect. It is reported that flavonoieds act on GABAergic system in the brain. Flavonoieds can also enhance the effect of benzodiazepines on GABA receptors (62). Other components of essential oil of *R. damascena* such as geraniol and eugenol have been shown to have antiepileptic effect (65). However, the exact mechanistic effect of these compounds is unknown. 

The effects of the essential oil of *R. damascena* as an adjunct in treatment of children with refractory seizures were also studied and showed a significant reduction in the mean frequency of seizures in patients using essential oil of the plant. Therefore, the essential oil of *R. damascena* has beneficial antiepileptic effect in children with refractory seizures (64).

***Effect on respiratory system***

Research about respiratory effect of *R. damascena* is sparse and only our laboratory evaluated this effect. We showed that the ethanolic and aqueous extracts of this plant significantly reduce number of coughs induced by citric acid, in guinea pigs (46). In another study the effect of ethanolic extract and essential oil on tracheal smooth muscle of guinea pigs contracted by KCl and methacholine were studied. The results showed a potent relaxant effect of extract and essential oil that was comparable to that of theophylline (66). The exact mechanism(s) of antitussive effect of* R. damascena* is (are) not clarified. However, this effect of *R. damascena* might be due to its possible tachykinin inhibitory substance(s) content mediating both bronchodilatory and antitussive effects (67). 

The mechanism(s) of relaxant effect of *R. damascenea *on tracheal smooth muscle of guinea pigs is (are) unknown. This effect may be produced by several different mechanisms. Because the relaxant effect of adrenoceptors on guinea pig airway and bronchodilatory effect of H_1_ blocking drugs have been shown previously (68-69), we suggested that some components of this plant can stimulate β-adrenergic receptors or inhibit histamine (H_1_) receptors. In fact, the extract and essential oil from *R. damascene *did not show any significant relaxant effect on incubated tracheal chains with β-adrenergic and H_1_ receptors antagonists. These results indicated a stimulator effect for this plant on β-adrenoceptors and/or histamine (H_1_) receptors blocking effect. Based on bronchodilatory effect of calcium channel blockers, an inhibitory effect of this plant on calcium channels of guinea pig tracheal chain also suggested (46,66).

The aqueous, ethyl acetate and n-butanol fractions of *R. damascena* also showed relaxant effect on tracheal smooth muscle of guinea pigs (70). The results of this study also showed more potent relaxation effect of ethyl acetate fraction on tracheal smooth muscle compared to theophylline, while effect of aqueous and n-butanol fraction was relatively weak. The greater relaxant effect of ethyl acetate fraction compared to the other two fractions suggests that lipid soluble (non-polar) constituents of this plant are mainly responsible for its relaxant effect on tracheal smooth muscle. The results also suggest an inhibitory effect of aqueous and acetyl acetate fractions on muscarinic receptors (70). The effect of essential oil, extracts and fractions of the plant are summarized on Table 2. 

**Table 1.** Pharmacological effects of flowers from *Rosa damascene*

Type of solution	Effect	Method of study	Reference
Extract( ethanolic , aqueous)	Hypnotic	Pentobarbital-induced sleep time	43, 44
Fraction(ethyl acetate, aqueous, n-butanol)			
Extract (Hydroalcoholic , ethanolic )	Analgesic	Hot plate , tail flick, acetic acid and formalin tests	49, 50
Essential oil	Anticonvulsant	Pentylenetetrazole and kindling methods	62, 63
Ethanolic and aqueous extracts	Antitussive	Citric acid method	46
Ethanolic extract , essential oil	Bronchodilatory	Tracheal chains	66, 70
Fraction(ethyl acetate, aqueous , n-butanol)			
Aqueous-ethanolic extract	Potentiation of HR and contractility	Isolated heart (Langendorff mode )	71
Compounds purified from the methanol extract	Anti-HIV	Effect on C8166 and H9 cells infected with HIV	33
Essential oil and absolute extract	Antibacterial	Disk method, well-diffusion , microdilution method	33, 77, 78
Methanol extract	Anti-diabetic	Measurement of α-glucosidase activity	73
Extract (hydroalcohlic, ethanolic, fresh	Antioxidant	Measurement of free-radical-scavenging activity	76, 83, 84
flower, spent flower),essential oil			
Boiled extract	Laxative and prokinetic	Frequency of defecation, Intestinal transit time	78
Hydroalcoholic extract	Anti-inflammatory	Rat paw edema induced by carrageenan	85

**Table 2.** Relaxant effect of extract, essential oil and fractions from *Rosa damascena* in comparison with negative control (saline) and positive control (theophylline) in group 1 experiments (KCl) (66, 67, 70).

Different Solution	Concentration	G1	G2	G3
	0.25	5.60±2.42	1.62±1.16	0.00±0.00
Ethanolic extract	0.50	11.60±4.95	17.12±4.06	0.00±0.00
	0.75	20.00±9.12	43.25±6.32	4.00±3.00
	1.0	41.60±11.95	60.37±6.98	9.00±5.00
	0.25	22.80±6.38	15.19±1.57	0.00±0.00
Essential oil	0.50	32.40±7.36	38.50±4.25	2.00±2.00
	0.75	53.80±7.91	59.13±7.47	0.00±0.00
	1.0	82.40±7.92	67.88±6.27	8.00±5.00
	0.1	-3.50±1.17	18.25±2.40	-
Aqueous F.	0.2	-6.30±0.50	26.75. ±3.32	-
	0.4	-6.60±0.98	34.88±4.37	-
	0.1	33.80±2.13	21.50±5.37	-
Ethyl acetate F.	0.2	48.20±3.50	44.81±11.55	-
	0.4	68.42±4.48	77.89±9.14	-
	0.1	3.48±1.20	1.56±0.87	-
N-buthanol F.	0.2	6.20±0.46	3.50±0.87	-
	0.4	24.00±3.77	5.00±1.17	-
	0.25	-4.36±2.44	-1.92±0.27	-
Theophylline	0.50	17.81±7.44	12.43±1.63	-
	0.75	50.40±6.86	33.26±3.02	-
	1.0	88.20±7.28	73.81±4.53	-

Values are presented as mean±SEM. The unit of concentration for essential oil was vol%, for extract and fractions was g%, and for theophylline was mM. Group 1 (G1); KCl induced contraction on non - incubated tracheal chains (n= 5), Group 2 (G2); methacholine induced contraction on non - incubated tracheal chains (n= 8) and Group 3 (G3); methacholine induced contraction on incubated tracheal chains of guinea pig with propranolol and chlorpheniramine (n= 5).

***Effect on cardiovascular***

The research on the cardiovascular effect of *R. damascena* is little. In one study aqueous-ethanolic extract from *R. damascena* potentially increased heart rate and contractility in isolated guinea pig heart. The mechanisms of these effects are unknown. However, a possible stimulatory effect of the plant on β-adrenoceptor of isolated guinea pig heart is suggested (71). 

Recently, a new compound named cyanidin-3-O-β-glucoside was isolated from the buds of *R. damascenea. *This compound can significantly suppressed angiotensin I-converting enzyme (ACE) activity. Because ACE is a key enzyme in production of angiotensin II, *R. damascena* may be effective to improve the cardiovascular function (72).

***Anti-HIV effects***

The effect of water and methanol extracts of *R. damascena* on HIV infection were studied *in vitro* (33). In this study, anti-HIV activities of the nine compounds including a new compound 2-phenylethanol-O-(6-O-galloyl)-β-D-glucopyranoside which were purified from the methanol extract were evaluated on C8166 human T lymphoblastoid cells infected with HIV-1MN and H9 human T-cell lymphoma cells chronically infected with HIV-1IIIB. Kaempferol 1 and its 3-O-β-D-glucopyranosides 3 and 6 exhibited the greatest activity against HIV infection of C8166 cells, whereas kaempferol-7-O-β-D-glucopyranoside showed no effect. Similarly, quercetin-7-O-β-D-glucopyranoside was inactive compared to quercetin 2. Compound 8, a new natural product exhibited some anti-HIV activity, presumably due to the presence of the galloyl moiety since 2-phenylethanol-O-β-D-glucopyranoside was inactive. In this study, authors compared the anti-HIV activities of the nine compounds and showed that the activity of the crude extract is due to the combined effects of different compounds acting additively against different stages of virus replication (33). 

***Anti- diabetic effect***

It has been found that* R. damascena* exert an anti-diabetic effect. Oral administration of the methanol extract of this plant significantly decreased blood glucose after maltose loading in normal and diabetic rats in a dose- dependent manner. In addition, its methanol extract inhibited postprandial hyperglycemia similar to of acarbose. It was found that *R. damascena* is a potent inhibitor of α-glucosidase enzyme (73). Therefore, anti-diabetic effect of this plant maybe mediated by inhibition of α-glucosidase that suppressed carbohydrate absorption from the small intestine and can reduce the postprandial glucose level (74).

***Antimicrobial effects***

It has been shown that *R. damascena* has wide spectrum antimicrobial activities. Essential oil, absolute and hydrosol are important products that showed these effects.

Ulusoy *et al *(2009) showed that essential oil and absolute have strong antibacterial activity against *Escherichia coli*, *Pseudomonas aeruginosa*, *B. subtilis*, *Staph. aureus*, *Chromobacterium violaceum* and *Erwinia carotovora* strains. The *C. violaceum *was the most sensitive microorganism against rose essential oil and absolute. *E. coli* was also sensitive against rose essential. However, hydrosol had no antimicrobial activity against any of the microorganisms (35). Rose absolute also showed antibacterial activity against both gram-negative and gram-positive bacteria (35). 

In other study, the essential oil of *R. damascena* petals was evaluated for its antibacterial effects against three strains of *Xanthomonas axonopodis* spp. vesicatoria. The essential oil of *R. damascena* flower remarkably inhibited the growth of the tested strains of *X. axonopodis* vesicatoria (75). Antibacterial activity of the both fresh flower (FF) and spent flower (SF) extracts of *R. damascena* flower against 15 species of bacteria: *Aeromonas hydrophila, B. cereus, Enterobacter aerogenes, Enterococcus feacalis, E. coli, Klebsiella pneumoniae, Mycobacterium smegmatis, Proteus vulgaris, Ps. aeruginosa, Ps. fluorescens, Salmonella enteritidis, Salmonella typhimurium, Staph. aureus, and Yersinia enterocolitica* were studied. Both extracts were effective against all the bacteria except *E. coli*, although the FF extract was more effective than the SF extract. FF and SF extracts showed the strongest effects against *S. enteritidis* and *M. smegmatis*, respectively (76).

The* in vitro* antibacterial activities of essential oil from* R. damasce* were also shown by disk diffusion testing *against E. coli, Staph. aureus and Ps. aeruginosa. R. damascena* showed antimicrobial activity against *Staph. aureus *in this study (77). 

The interaction between water extracts of *Psidium guajava*, *Rosmarinus officinalis*, *Salvia fruticosa*, *Majorana syriaca*, *Ocimum basilucum, Syzygium aromaticum*, *Laurus nobilis, *and *R. damascena *using both well-diffusion and microdilution methods against five *Staph. aureus *isolates; one Methicillin-resistant *Staph. aureus *(MRSA) and four Methicillin-sensitive *Staph. aureus *(MSSA) was studied. The results showed that synergism effect between antimicrobial agents and plant extracts was occurred in both sensitive and resistant strains but the magnitude of minimum fold inhibition in resistant strains especially MRSA strain was higher than the sensitive strains (78).

Essential oils of several plants including *R. damascena* were also tested for antimicrobial activity against gram-positive *Staph. aureus* (ATCC 25923), gram-negative *E. coli* (ATCC 25922), gram-negative *Ps. aeruginosa* (ATCC 27853), and yeast *Candida albicans* (ATCC 14053). The tested essential oils exhibited inhibitory and bactericidal activities against all tested microorganisms at low concentrations (79).

 Antibacterial effect of major components of rose oil (citrenellol, geraniol and nerol) was reported (77,80). Therefore, Antibacterial effect of rose oil maybe mediated by these components. Antibacterial properties of rose absolute could be attributed to its high phenylethyl alcohol content. The antimicrobial properties of alcohols have been known for a long time (81).

***Antioxidant effects***

The *R. damascena* similar to many aromatic and medicinal plants exhibits antioxidant properties. Sources of natural antioxidant are primarily phenolics compound that are found in all parts of plants such as the fruits, vegetables, seeds, leaves, roots and barks (82). The presence of phenolic compound in ethanolic extract of *R. damascena* has been shown by Kumar *et al *(2009). They determined antioxidant activity of this extract compare to standard antioxidant L-ascorbic acid by 1, 1-diphenyl-2-picryl hydrazyl (DPPH) free-radical method. This study showed that *R. damascena* has high antioxidant activities (83). The antioxidant activity of hydro-alcoholic extract of petals and essential oil of this plant was also evaluated by DPPH for measurement of free radical scavenging activity and by ferric ammonium thiocyanate method for evaluation of lipid peroxidation properties. Additionally, three flavonol glycosides of ethanolic extract including quercetin-3-O-glucoside, kaempferol-3-O-rhamnoside and kaempferol-3-O-arabinoside have antioxidant activity. However, the potential of this effect is maybe due to existence of quercetin 3-O-glucoside and other flavonoids in the extract (9). Both fresh flower (FF) and spent flower (SF) extracts of *R. damascena* flowers also showed antioxidant activity. However, the antioxidant activity of FF extract was higher than that of SF extract (76). The antioxidant effect of *R. damascene* and its inhibitory effect on lipid oxidation were evaluated in an *in vivo* study. The results showed a potent antioxidant and lipid peroxidation inhibitory effects comparable to -tocopherol and suggest that the plant can be considered as a medical source for the treatment and prevention of many free radical diseases (84). 

***Other effects***

*The anti-inflammatory effect*

This plant has also been shown to have anti-inflammatory effect (85). The effect of essential oil and hydroalcoholic extract of *R. damascena* on rat paw edema induced by carrageenan was demonstrated. Essential oil had no anti-inflammatory effect while the extract could significantly reduce edema which maybe acted by inhibiting the mediators of acuteinflammation (85,86). In addition,* R. damascena *contains vitamin C (13) which has antioxidant and anti-inflammatory effects (50,86).

*The laxative and prokinetic Effects*

Similar to traditional medicine gavage of boiled extract of *R. damascena *in rats showed significant laxative effects (increasing feces water content and the frequency of defecation). Because intraperitoneal (i.p.) injection of extract showed symptoms of constipation (no feces in 24 hr), it seems the laxative effects is partly due to osmotic infiltration of fluids into intestinal lumen (87). 

*Protective effect* *against surgically induced reflux esophagitis*

The effect of poly herbal formulation (PHF) consisting of seven medicinal plants namely *Aegle marmelos*, *Elettaria cardamomum*, *Glycyrrhiza glabra*, *Citrus aurantifolia*, *R. damascena*, *Cissus quadrangularis, *and *Saccharum officinarum* on experimentally induced reflux esophagitis and gastrointestinal motility in animals was also evaluated. The PHF exhibited significant decrease in lesion index and enhance the % protection of lesion in experimentally induced reflux esophagitis. The study indicated that the PHF has protective effect against surgically induced reflux esophagitis which may be due to its gastro protective, anti-oxidant, and prokinetic activity (88).

*Anti-aging effects*

The effects of a rose-flower extract on the mortality rate of *Drosophila melanogaster *was evaluated in a recent study. Supplementing *Drosophila *with the plant extract resulted in a statistically significant decrease in mortality rate in male and female flies. Moreover, the observed anti-aging effects were not associated with common confounds of anti-aging properties, such as a decrease in fecundity or metabolic rate. Therefore, *R. damascena *can extend *Drosophila *life span without affecting physiological mechanisms. This study postulated that the plant’s antioxidant properties could have contributed to prolongation of life span in *Drosophila* (89).

*The anti- lipase effect*

In a recent study, the anti-lipase effect of the extract of several plant including *R. damascena* was studied. The ethanolic extract of *R. damascena* in this study showed anti lipase effect (90).

*Ophthalmic effect*

The effect of a herbal eye drop preparation (Ophthacare^®^) containing different herbs including *R. damascena* in patients suffering from various ophthalmic disorders namely, conjunctivitis, conjunctival xerosis (dry eye), acute dacryocystitis, degenerative conditions (pterygium or pinguecula), and postoperative cataract patients was studied. These herbs have been conventionally used in the Ayurvedic system of medicine since time immemorial and reportedly possess anti-infective and anti-inflammatory properties. An improvement was observed after receiving the herbal eye drop treatment in most of the cases. These results showed that herbal eye drop, Ophthacare^®^, has a useful role in a variety of infective, inflammatory and degenerative ophthalmic disorders (91).

## Conclusion

The *R. damascena* is one of the most important species of Rosaceae family mainly known for its perfuming. Its major products are rose water and essential oil. 

This plant contains several components such as terpenes, glycosides, flavonoids, and anthocyanins that have beneficial effects on human health. The pharmacological effects of *R. damascene* are widespread. Most of the CNS effects are hypnotic, analgesic, and anticonvulsant effects. The respiratory, cardiovascular*,* laxative, antidiabetic, antimicrobial, anti-HIV, anti-inflammatory, and antioxidant are other effects of this plant. It is suggested that lipid soluble (non-polar) constituents of this plant are mainly responsible for most of the above-mentioned effects.
